# Significance of Semiquantitative Assessment of Preformed Donor-Specific Antibody Using Luminex Single Bead Assay in Living Related Liver Transplantation

**DOI:** 10.1155/2013/972705

**Published:** 2013-05-29

**Authors:** Atsushi Yoshizawa, Hiroto Egawa, Kimiko Yurugi, Rie Hishida, Hiroaki Tsuji, Eiji Ashihara, Aya Miyagawa-Hayashino, Satoshi Teramukai, Taira Maekawa, Hironori Haga, Sinji Uemoto

**Affiliations:** ^1^Department of Surgery, Graduate School of Medicine, Kyoto University, Kyoto 606-8507, Japan; ^2^Department of Surgery, Institute of Gastroenterology, Tokyo Women's Medical University, 8-1 Kawada-cho, Shinjuku-ku, Tokyo 162-8666, Japan; ^3^Department of Transfusion Medicine and Cell Therapy, Kyoto University Hospital, Kyoto 606-8507, Japan; ^4^Department of Diagnostic Pathology, Kyoto University, Kyoto 606-8507, Japan; ^5^Division of Clinical Trial Design and Management, Translational Research Center, Kyoto University Hospital, Kyoto 606-8507, Japan

## Abstract

*Aim*. To analyze the risks of preoperatively produced donor-specific antibody (DSA) in liver transplantation. *Methods*. DSA was assessed using direct complement-dependent cytotoxicity (CDC) and anti-human globulin- (AHG-) CDC tests, as well as the Luminex Single Antigen assay. Among 616 patients undergoing blood type identical or compatible living donor liver transplantation (LDLT), 21 patients were positive for CDC or AHG-CDC tests, and the preserved serum from 18 patients was examined to determine targeted Class I and II antigens. The relationships between the mean fluorescence intensity (MFI) of DSA and the clinical outcomes were analyzed. *Results*. Patients were divided into 3 groups according to the MFI of anti-Class I DSA: high (11 patients with MFI > 10,000), low (2 patients with MFI < 10,000), and negative (5 patients) MFI groups. Six of 11 patients with high Class-I DSA showed positive Class-II DSA. Hospital death occurred in 7 patients of the high MFI group. High MFI was a significant risk factor for mortality (*P* = 0.0155). Univariate analysis showed a significant correlation between MFI strength and C4d deposition (*P* = 0.0498). *Conclusions*. HLA Class I DSA with MFI > 10,000 had a significant negative effect on the clinical outcome of patients with preformed DSA in LDLT.

## 1. Introduction

The effect of preformed antibodies targeting human leukocyte antigens (HLAs) on the outcome of organ transplantation has been demonstrated in kidney, heart, and lung transplantation, and a lymphocyte crossmatch test (LCT) is considered mandatory. On the other hand, such effect is controversial in liver transplantation, even in living donor liver transplantation (LDLT) [1, 2]. We previously reported the negative effect of preformed donor-specific antibody (DSA) in LDLT and demonstrated that the risk factors for mortality were an adult recipient and a female gender [2, 3]. However, our previous studies did not include determination of the HLA targeted by the preformed DSA as well as analysis of the relationship between the amount of HLA-specific DSA and clinical outcome. Recently, a single antigen bead assay using the Luminex analyzer has enabled the determination of targeted HLA [4]. Furthermore, the mean fluorescence intensity (MFI) generated by the Luminex analyzer might enable the measurement of the amount of DSA. Musat et al. reported the significance of DSA in rejection after liver transplantation using the Luminex analyzer and the histological examination of C4d deposition [5]. 

In the present study, we retrospectively analyzed the targeted HLA and the amount of DSA in the preserved serum of patients with a positive LCT prior to LDLT to clarify the relationship between the amount of DSA and the clinical outcome. 

## 2. Methods

### 2.1. Patients

Between January 2000 and March 2008, 616 patients underwent blood type identical or compatible LDLT. Among them, 21 recipients (3.4%) were LCT positive preoperatively. Pretransplant sera from 18 of these 21 recipients were preserved and available for examination in the present study. These 18 patients were enrolled in this study and their characteristics are shown in Table 1. Their ages ranged from 6 months to 67 years (median, 48.0 years). There were 2 men and 16 women, 10 of whom had a history of pregnancy. Eleven patients had histories of blood transfusion, 5 had none, and 2 had no record. Nine patients had upper abdominal surgeries possibly leading to operative difficulty in 11 patients with histories of abdominal surgeries. In 2 patients, the second transplantations were investigated. The donors were 6 parents, 6 sons or daughters, 2 siblings, and 4 unrelated spouses.

This study was approved by the Ethics Committee of Kyoto University Hospital according to the Declaration of Helsinki of 1975 as revised in 2008.

### 2.2. Liver Transplantation and Initial Immunosuppression

All recipients underwent LDLT employing our standard methods. In our protocol, the target trough level of tacrolimus was 10 to 15 ng/mL for the first 2 weeks, and then it was tapered and adjusted individually depending on each patient's condition [6]. Intravenous methylprednisolone was the initial steroid used immediately after reperfusion, which was tapered, and then followed by oral prednisolone on day 8, which was stopped at 3 months. 

### 2.3. Histological Evaluation

Liver specimens were fixed in 10% buffered formalin, processed routinely, and cut into 3 *μ*m thick paraffin sections. The routine staining methods included hematoxylin and eosin, Masson trichrome, and cytokeratin 7 (CK-7, OV-TL 12/30, Dako, Denmark; dilution 1 : 200) staining. Acute cellular and chronic rejections were evaluated according to the Banff Schema [7]. Each evaluation was blindly conducted by 2 pathologists (A. Miyagawa-Hayashino and H. Haga).

### 2.4. C4d Staining

Polyclonal antibody against C4d complement (BI-RC4D; Biomedia, Vienna, Austria; dilution 1 : 50) was used for immunostaining with an automated immunostainer (BENCHMARK XT, Ventana Medical Systems, Tucson, AZ). For antigen retrieval, deparaffinized and rehydrated sections were treated with protease I (Ventana Medical Systems; 0.5 U/mL) at 37°C for 20 minutes [8, 9]. 

Biopsy specimens in which only the vascular endothelium was stained were evaluated as endothelial positive. Biopsy specimens in which both the vascular endothelium and the stroma were stained were evaluated as endothelial and stromal positive (E&S positive). Any C4d staining on elastic fibers within the arteries and stroma was regarded as a nonspecific finding without clinical significance [10].

### 2.5. Lymphocyte Crossmatch Test

Pretransplant LCT was performed using both direct complement-dependent cytotoxicity (CDC) and CDC with added anti-human globulin (AHG-CDC) tests. Incubation was conducted using 1 milliliter of donor lymphocyte suspension and 5 milliliters of recipient serum in a Terasaki plate (Nunc, Roskilde, Denmark) at room temperature for 30 min. In the AHG-CDC test, AHG (Goat IgG k and l light chains) was added and incubated at room temperature for 3 min. Five microliters of rabbit complement were added to each well and the mixture was incubated at room temperature for 60 min. Two microliters of 5% eosin solution were added and the mixture was examined using phase-contrast microscopy (IMT-2; Olympus, Tokyo, Japan). The results were considered positive when more than 20% of the donor lymphocytes were killed by the recipient's serum in either test. Dithiothreitol was not used for the inactivation of IgM antibodies.

### 2.6. HLA DNA Typing

Tissue typing was performed in patients and donors for HLA-A, HLA-B, HLA-C, HLA-DR, and HLA-DQ for class I and II loci using WAKFlow (Wakunaga Corp., Hiroshima, Japan) and Luminex xMAP technology (Luminex Corp., Austin, TX) [11].

### 2.7. Antibody Screening Employing LABScreen Mixed Assay

Pretransplant sera were retrospectively analyzed for HLA antibodies employing a multiplexed microsphere-based suspension array from Luminex xMAP technology (Luminex Corp.). In brief, 5 microliters of LABScreen Mixed (One Lambda, Canoga Park, CA) color-coded microbeads coated with purified HLA were incubated in the dark for 30 min at 20°C to 25°C with 20 microliters of test serum. Any HLA antibodies present in the sera were bound to the LABScreen Mixed surface antigens coating the microbeads and were subsequently labeled with R-phycoerythrin-conjugated goat anti-human IgG. The microbead fluorescent emission of R-phycoerythrin was then detected and quantified using the LABScan 100 flow analyzer (One Lambda).

The determination of positive and negative sera was performed with One Lambda software (LABScreen PRA software, One Lambda) according to the manufacturer's protocol. Sera reactivity was assessed based on the fluorescent signal for each HLA-coated microbead following correction for nonspecific binding to the negative control microbead. In the LABScreen Mixed assay, the normalized fluorescent signal is equal to the value of the antigen-coated microbead minus the value of the negative control microbead. If any one microbead in the mixed assay is positive, the result is considered positive. 

### 2.8. Single Antigen Bead Assay

The Single Antigen bead assay is essentially the same as the assay outlined earlier according to manufacturer's protocol. In brief, 20 microliters of test serum were incubated with 5 microliters of the selected single beads and 5 microliters of LABScreen Singles control beads. Samples were read on the LABScan 100 flow analyzer (One Lambda). Raw trimmed MFIs were obtained from the output file generated by the flow analyzer and normalized.

### 2.9. Statistical Analysis

A *P* value < 0.05 was used for variable selection and was considered to indicate a statistically significant difference. SAS version 9.2 (SAS Institute Inc., Cary, NC) was used for statistical analysis. The log rank test was employed to estimate significance. 

## 3. Results

### 3.1. Patient Survival

**Figure 1:**
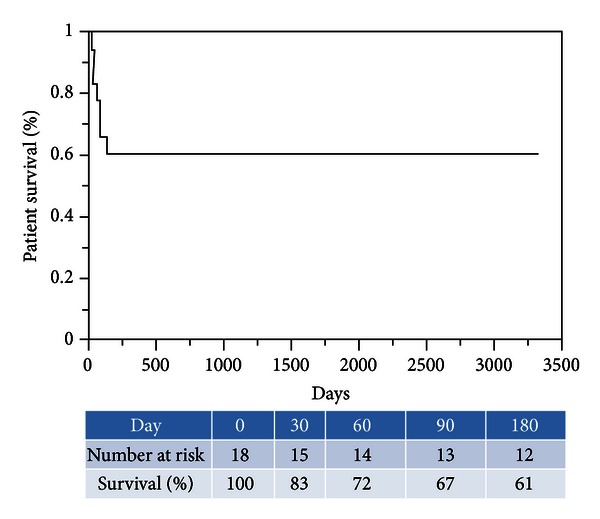
Patient survival curve.

The patient survival rate was 72% on postoperative day (POD) 60, 67% on POD 90, and 61% on POD 180 until 10 years (Figure 1).

### 3.2. Lymphocyte Crossmatch Test and Donor-Specific Antibody

Results of the CDC and AHG-CDC tests, the LABScreen Mixed assay (One Lambda), and LABScreen Single Antigen assay (One Lambda) are shown in Table 2.

Four patients showed both positive CDC and AHG-CDC tests, 10 patients showed negative CDC and positive AHG-CDC tests, and 4 patients showed positive CDC and negative AHG-CDC tests. Based on the Mixed assay, 15 patients had anti-HLA Class I antibodies. Based on the Single Antigen assay, DSA was detected in 13 out of the 18 patients and non-DSA was detected in 15 patients. When a patient showed positive DSA or non-DSA against more than 2 HLAs of donor or non-donor, the highest value was selected as “the peak value.” The MFI peak value in each patient ranged from 571 to 20,259 (median, 15,864) in 13 DSA-positive patients and 901 to 20,576 (median, 14,399) in 15 non-DSA-positive patients. Based on receiver operating characteristic (ROC) curve, the point with the best sensitivity and specificity for mortality was 12,211 in patient 4. The area under the curve was 0.792. The next small value was 8,272 in patient 12. The 18 patients were divided into 3 groups: high (MFI > 10,000; *n* = 11), low (MFI < 10,000; *n* = 2), and negative (DSA not detected; *n* = 5). Two patients showed negative DSA in the 15 patients with positive non-DSA.

Among 7 patients who had anti-HLA Class II antibodies based on the Mixed assay, DSA was positive in 6 patients based on the Single Antigen assay. All 6 patients presented positive Class I DSA. The MFI peak value in each patient ranged from 2,793 to 18,760 (median, 8,776) in 6 DSA-positive patients. 

Regarding the relationship between the LCT and the Single Antigen assay, all 4 patients with positive CDC and AHG-CDC tests had DSA with high MFI. Ten patients with negative CDC and positive AHG-CDC tests consisted of patients from the 3 groups (7 high MFI, 2 low MFI, and 1 negative DSA). Four patients with positive CDC and negative AHG-CDC tests showed negative DSA on the Single Antigen assay.

Regarding the relationship between high DSA or high non-DSA and possible backgrounds, there was no significant relationship between history of blood transfusion and high DSA (*P* = 0.306) and between history of blood transfusion and high non-DSA (Fisher exact test, *P* = 0.464). On the other hand, there was a significant relationship between history of pregnancy and high DSA (Fisher exact test, *P* = 0.003).

### 3.3. Histological Examination

The MFI of DSA, the histological findings of the first liver biopsy after transplantation, and the clinical outcomes of the 18 patients are shown in Table 2. Twelve patients underwent liver biopsy after transplantation: 9 within 90 days and 3 after 90 days. The major histological diagnosis was cholangitis in 5 patients, as reported by Takaya et al. [12]. 

 Eight of 12 initial biopsy specimens showed positive C4d staining: stromal and endothelial deposition in 4 patients and endothelial deposition in 4 patients. All 4 cases with endothelial C4d staining only showed focal staining (portal C4d immunolabeling of fewer than 50% of portal tracts). All 4 cases with endothelial and stromal C4d staining showed diffuse staining pattern (C4d deposition in the hepatic artery, portal vein, or capillary endothelium of more than 50% of portal tracts). There were cases showing sinusoidal C4d staining. Three of the 4 patients with stromal and endothelial deposition (75%) and 3 of the 4 patients with endothelial deposition (75%) showed positive DSA with high MFI. All C4d-negative patients showed negative DSA on the Single Antigen assay. A significant correlation between MFI strength and C4d deposition was found on univariate analysis (*P* = 0.0498). Two patients with negative DSA and positive non-DSA showed negative C4d staining.

### 3.4. Clinical Courses and Risk Factors of Mortality

Seven patients died within 4 months after transplantation. The causes of death were sepsis in 5 and vascular complications in 2. 

All of the 7 patients who died early had DSA with high MFI prior to LDLT. The risk factors for mortality were analyzed and a high level of Class-I DSA was found to be a significant risk factor (Fisher exact test, *P* = 0.015) (Table 3). The 1-year patient survival rate was 36% in the high MFI DSA group and 100% in the low and negative MFI DSA groups (Log-rank test, *P* = 0.042). Non-DSA or Class-II DSA was not a significant risk factor. History of blood transfusion and histories of abdominal surgery were not either. 

Eleven patients are alive and the follow-up period ranged from 777 to 3,479 days. All but one showed normal hepatic chemistries and their performance status was 0. Case 2 showed an AST of 21 U/L, an ALP of 2,140 U/L, and a total bilirubin level of 4.4 mg/dL, and her performance status was 2 at 3,479 days after transplantation. Histological findings from the last liver biopsy specimens on postoperative days (PODs) 339 to 2,360 of 9 out of 11 alive patients are shown in Table 2. Three patients showed mild portal inflammation, 1 cholangitis with possible recurrence of primary biliary cirrhosis, 1 cholangitis with recurrence of hepatitis C, 1 resolving cholangitis, 1 acute cellular rejection, 1 steatohepatitis, and 1 no remarkable findings. 

## 4. Discussion

To the best of our knowledge, this is the first study to clarify the relationship between LCT and Single Antigen assay in liver transplantation with the following important findings. First, both the positive CDC and AHG-CDC tests indicated a high level of DSA against HLA. Second, the positive AHG-CDC test indicated the presence of DSA against HLA. Third, the positive CDC test with negative AHG-CDC test indicated contribution of IgM-DSA. Based on these findings, we deduced that the AHG-CDC test is not sufficiently sensitive to predict postoperative mortality but could indicate the presence of a high level of DSA against Class I. 

Castillo-Rama reported the significance of the Mixed assay in liver transplantation [13]. The Mixed assay is a screening system that detects the presence of antibodies against Class I or II but does not show the specificity of DSA. In this study, 2 patients with a positive Mixed assay against Class I had no DSA. The Mixed assay is useful for identifying patients requiring the Single Antigen assay from the viewpoint of cost reduction. However, the Single Antigen assay is the optimal method for the identification and quantitative analysis of DSA in liver transplantation. 

The incidence of preformed DSA is approximately 10% in deceased donor liver transplantation in Western counties [12, 13]. The incidence in this LDLT series including pediatric patients was 3.4% (21 in 616 patients), whereas that in the Ashihara series consisting of adult patients in our center was 3.0% [2]. With the assumption that the sensitivity of LCT is the same, recipients in Western countries show a higher chance of developing preformed DSA based on LCT. In the present study, a significant relationship between history of pregnancy and high DSA was found. The chance of husbands, sons, and daughters becoming donors in LDLT for female patients was high, but this could lead to unfavorable outcomes owing to a large amount of DSA secondary to strong sensitization during their pregnancy. Therefore, information on the specificity and amount of DSA is very important in LDLT.

All DSA-positive patients showed positive non-DSA. It can be considered that positive DSA is part of the phenomena of sensitization against HLA including donor-specific antigens. However, in this study, high DSA positivity was found to be a significant risk factor for mortality. Blood transfusion is theoretically the most important contributing factor for sensitization. However, blood transfusion was found to be independent of the high positivity of DSA and non-DSA. Therefore, we analyzed the combined effect of high DSA or high non-DSA and history of blood transfusion. A significant difference in the incidence of mortality between positive history of blood transfusion and high DSA (*n* = 7, 6/7) and others (*n* = 9, 0/9) was found (Fisher exact test, *P* = 0.001). Moreover, there was a significant difference in the incidence of mortality between positive history of blood transfusion and high non-DSA (*n* = 8, 6/8) and others (*n* = 8, 0/8) (Fisher exact test, *P* = 0.007). Taken together, when patients who had histories of blood transfusion were highly sensitized, the mortality increased. This phenomenon might be related to unfavorable immune regulation leading to postoperative fatal infections. 

Based on these results, we changed our policy of donor selection. A donor candidate to whom a recipient is highly sensitized with MFI > 10,000 is rejected. A donor to whom a recipient is sensitized with MFI < 10,000 is not considered when other donors are available; however, such a donor can be accepted after B cell desensitization using our protocol for ABO incompatible transplantation, which involves administration of rituximab, plasma exchange, and intravenous immunoglobulin. During 1 year from December 2009, 100 patients were evaluated regarding their LDLT and 12 patients were found to be positive for anti-class I antibodies. Five of them had DSA against donor candidates. Only 1 patient was highly sensitized and another family member to whom the patient had no DSA donated the graft. Another patient could change the donor, but the remaining 3 could not. All 5 patients survived after the transplantation.

A limitation of this study is that it did not reveal the relationship between the specific HLA and the clinical outcome in recipients with preformed DSA. Although the significance of MFI generated by the Luminex analyzer for the DSA assay has not yet been established, this study showed that DSA-MFI > 10,000 had a significant effect on the clinical outcome and a significant relationship with LCT. Further studies to clarify the meaning of low MFI and postoperative changes in DSA using the Single Antigen assay are required.

## Figures and Tables

**Table 1:** Profiles of crossmatch-positive recipients.

Patient no.	Primary disease	Age (years)	Sex	Donor	Blood type compatibility	Graft type	GRWR (%)*	History of pregnancy	History of blood transfusion	History of abdominal surgery
1	PBC^†^	67	F	Son	Identical	Left lobe	1.43	Yes	Yes	Cholecystectomy, Hassab operation
2	PBC	47	F	Son	Compatible	Right lobe	1.29	Yes	Yes	Cholecystectomy, choledocojejunostomy, hepaticojejunostomy
3	Biliary atresia	19	F	Mother	Identical	Right lobe	1.16	No	Yes	Kasai operation
4	HCV-LC^‡^	49	F	Husband	Identical	Right lobe	1.41	Yes	Yes	Appendectomy
5	PBC	53	F	Daughter	Identical	Right lobe	0.98	Yes	Unknown	No
6	HBV-FHF^$^	62	F	Daughter	Identical	Left lobe	0.77	Yes	No	No
7	Metastatic neuroendocrine tumor of pancreas	47	F	Husband	Compatible	Left lobe	1.00	Yes	Yes	Distal pancreatectomy and splenectomy and repair of portal vein injury
8	HCV-LC	53	F	Father	Identical	Right lobe	0.75	Yes	Unknown	Hysterectomy
9	Graft failure due to portal vein thrombosis after LDLT^*⋀*^	25	F	Sister	Identical	Right lobe	1.66	No	Yes	Kasai operation, splenectomy, distal splenorenal shunt, 1st LDLT
10	Congenital liver fibrosis	44	F	Husband	Identical	Right lobe	1.26	Yes	Yes	Splenectomy, esophageal transection, cholecystectomy, subtotal gastrectomy
11	LC	55	F	Son	Identical	Right lobe	1.16	Yes	No	No
12	PBC	50	F	Daughter	Identical	Right lobe	0.96	Yes	Yes	No
13	cryptogenic LC (AIH^#^)	51	F	Sister	Identical	Left lobe	0.72	No	Yes	No
14	Biliary atresia	0 (6 M)	F	Mother	Identical	Lateral segment	3.19	No	No	Kasai operation, revision
15	Biliary atresia	30	F	Mother	Identical	Left lobe	0.91	No	No	Kasai operation
16	HBV-LC, HCC**	50	M	Wife	Compatible	Right lobe	0.82	No	No	No
17	Wilson's disease	8	M	Mother	Identical	Lateral segment	1.34	No	Yes	No
18	Graft failure due to outflow block after LDLT	0 (11 M)	F	Mother	Compatible	Lateral segment	2.94	No	Yes	Kasai operation, 1st LDLT

*graft recipient weight ratio; ^†^primary biliary cirrhosis; ^‡^hepatitis C virus-related liver cirrhosis; ^$^hepatitis B virus-related fulminant hepatic failure; ^*⋀*^living donor liver transplantation; ^#^autoimmune hepatitis; **hepatocellular carcinoma.

**Table 2:** Results of lymphocyte crossmatch test, Mixed assay, and Single Antigen assay and histological findings and clinical outcomes in crossmatch-positive recipients.

Patient no.	LCT*		Mixed assay	MFI^$^ of anti-Class I HLA antibody		Mixed assay	MFI^$^ of anti-Class II HLA antibody		Histological findings of the first liver biopsy (POD)	C4d deposition	Outcome	Causes of death	Histological findings of the last liver biopsy in alive patients (POD)
	CDC^†^	AHG-CDC^‡^	HLA-Ab class I	DSA^*⋀*^	Non-DSA	HLA-Ab class II	DSA^*⋀*^	Non-DSA			Alive/dead (POD)		
1	**+**	**+**	**+**	High	High	−	Low	High	Cholangitis (40)	E^#^	Dead (118)	Sepsis	
2	**+**	**+**	**+**	High	High	**+**	High	High	Cholangitis (183)	E & S**	Alive (3479)		Chronic cholangitis, possible PBC^‡‡^ recurrence (2360)
3	**+**	**+**	**+**	High	High	**−**	Negative	Negative	No biopsy	**−**	Dead (28)	Hepatic artery rupture due to biliary leakage	
4	**+**	**+**	**+**	High	High	**+**	High	High	Cholestasis (18)	E & S	Dead (69)	Sepsis after repeated steroid pulse therapy for refractory ACR	
5	**−**	**+**	**+**	High	High	**+**	Low	Low	No biopsy		Dead (26)	Sepsis and fungal infection after steroid pulse therapy for refractory ACR	
6	**−**	**±**	**+**	High	High	**−**	Negative	Negative	No biopsy	**−**	Alive (1286)		No biopsy
7	**−**	**+**	**+**	High	High	**+**	Negative	Negative	Lobular inflammation (3)	E & S	Dead (49)	Intra-abdominal hemorrhage, sepsis after steroid pulse therapy for severe ACR	
8	**−**	**+**	**+**	High	High	**+**	High	Low	Acute cholangitis (18)	E	Alive (1160)		Chronic cholangitis, HCV^$$^ recurrence (339)
9	**−**	**+**	**+**	High	High	**−**	Negative	Negative	No biopsy	**−**	Dead (72)	Sepsis of intestinal perforation	
10	**−**	**+**	**+**	High	High	**+**	Low	Low	No biopsy	**−**	Dead (12)	Intra-abdominal hemorrhage and hepatic failure	
11	**−**	**+**	**+**	High	High	**−**	Negative	Negative	Steatosis (32)	E	Alive (1407)		Mild portal inflammation (1377)
12	**−**	**+**	**+**	Low	High	**+**	Negative	Negative	Cholangitis (183)	E & S	Alive (2660)		Resolving cholangitis (2286)
13	**−**	**+**	**+**	Low	Low	**−**	Negative	Negative	Steatosis (61)	E	Alive (777)		Steatohepatitis (613)
14	**−**	**+**	**+**	Negative	Low	**−**	Negative	Negative	Cholangitis (28)	N	Alive (1885)		Mild portal inflammation (913)
15	**+**	**−**	**+**	Negative	Negative	**−**	Negative	Negative	Cholestasis (20)	N	Alive (1490)		ACR (468)
16	**+**	**−**	**−**	Negative	Negative	**−**	Negative	Negative	No biopsy	**−**	Alive (1095)		No biopsy
17	**+**	**−**	**−**	Negative	Negative	**−**	Negative	Negative	Severe ACR^††^ (8)	N	Alive (3318)		No remarkable findings (720)
18	**+**	**−**	**−**	Negative	Low	**−**	Negative	Negative	Centrilobular hemorrhage and congestion (228)	N	Alive (2765)		Mild portal inflammation and fibrosis (2273)

*lymphocyte crossmatch test; ^†^direct complement-dependent cytotoxicity; ^‡^anti-human globulin with added CDC; ^$^mean fluorescence intensity (high, >10,000; low, <10,000); ^*⋀*^donor-specific antibody; ^#^endothelial deposition; **endothelial and stromal deposition; ^††^acute cellular rejection; ^‡‡^primary biliary cirrhosis; ^$$^hepatitis C.

**Table 3:** Risk factors for mortality.

Characteristics	Number	Mortality	
		%	*P* value*
Age			0.245
<18 years old	3	0	
≥18 years old	15	47	
History of pregnancy			0.367
No	8	25	
Yes	10	50	
History of blood transfusion			0.093
No	5	0	
Yes	11	55	
Unknown	2	—	
History of upper abdominal surgery			0.335
No	9	22	
Yes	9	56	
Donor selection			0.335
Husband-son-daughter	9	56	
Others	9	22	
Graft type			0.358
Right lobe	10	50	
Left lobe	5	40	
Lateral segment	3	0	
GRWR^†^			0.245
>0.8	15	47	
<0.8	3	0	
CDC^‡^			1.000
Negative	10	40	
Positive	8	38	
AHG-CDC^$^			0.119
Negative	4	0	
Positive	14	50	
Mix assay Class I			0.245
Negative	3	0	
Positive	15	47	
FI of anti-Class I-DSA^*⋀*^			0.015
Negative	5	0	
Low	2	0	
High	11	64	
FI of anti-Class I-non-DSA			0.110
Negative	3	0	
Low	3	0	
High	12	58	
Mix assay Class I			0.205
Negative	11	27	
Positive	7	39	
FI of anti-Class I-DSA^*⋀*^			0.057
Negative	12	25	
Low	3	100	
High	3	33	
FI of anti-Class I-non-DSA			
Negative			
Low			
High			
C4d staining			0.709
Negative	4	0	
Endothelial	4	25	
Endothelial and stromal	4	50	

*Fisher exact test; ^†^graft recipient weight ratio; ^‡^compliment-dependent cytotoxicity; ^$^anti-human globulin with added compliment-dependent cytotoxicity; ^*⋀*^fluorescence intensity of single antigen assay for anti-Class I donor-specific antibody (high, >10,000; low, <10,000).

## Abbreviations

DSA: Donor-specific antibody
LDLT: Living donor liver transplantation
HLA: Human leukocyte antigens
LCT: Lymphocyte cross-match test
CDC: Direct complement-dependent cytotoxicity
AHG: Anti-human globulin
MFI: Mean fluorescence intensity.
